# Comparison of portable devices with standard glaucoma diagnostic testing for the detection of glaucoma for the purposes of glaucoma case finding in low-and middle- income countries

**DOI:** 10.1038/s41433-026-04297-4

**Published:** 2026-02-23

**Authors:** Farouk Garba, Fatima Kyari, Matthew Burton, Victor H. Hu, David MacLeod, Peter Jones, Mubarak Bello, Abdulrashid Ibrahim, Goodness Diala, Ajefu Rose Ada, Kenneth Ezurike, Micheal Ochefu Ajefu, Abigail Yohanna, Winifred Nolan

**Affiliations:** 1 International Centre for Eye Health, Faculty of Infectious and Tropical Diseases, London School of Hygiene and Tropical Medicine, London, UK; 2 Department of Ophthalmology, Faculty of Clinical Sciences, College of Medical Sciences, Ahmadu Bello University, Zaria, Nigeria; 3 College of Health Sciences, University of Abuja, Abuja, Nigeria; 4 City St George’s, University of London, Department of Optometry & Visual Sciences, School of Health & Psychological Sciences, London, UK; 5 NIHR Moorfields Biomedical Research Centre, Moorfields Eye Hospital NHS Foundation Trust, London, UK

**Keywords:** Outcomes research, Epidemiology

## Abstract

**Background/objectives:**

Glaucoma is a leading cause of blindness worldwide, with the greatest burden in low- and middle-income countries (LMICs) where access to diagnostic services is limited. Portable devices, which are simple to use and comparable to standard tests, may strengthen early detection and referral. This study evaluated selected portable glaucoma assessment devices against standard reference tools in Nigeria and identified which tests or combinations most closely matched a glaucoma diagnosis.

**Subjects/methods:**

In this cross-sectional study at the University of Abuja Teaching Hospital, 312 participants (524 eyes) underwent both portable and conventional testing: Peek CS vs. Pelli-Robson (contrast sensitivity), Peek Acuity vs. LogMAR (visual acuity), iCare vs. Goldmann Applanation Tonometry (GAT) (intraocular pressure), Eyecatcher vs. Humphrey Field Analyzer (visual fields), and Remedio handheld fundus camera vs. slit lamp (optic discs). Agreement, diagnostic accuracy (AUC), completion rates, and patient preference were assessed.

**Results:**

Strong correlations were observed between iCare and GAT IOP (*r* = 0.96), Peek and LogMAR acuity (*r* = 0.82), and Remedio handheld fundus camera and slit lamp CDR (*r* = 0.82). The Remedio handheld fundus camera achieved the highest diagnostic accuracy (AUC = 0.91; sensitivity 82.4%, specificity 99.8%). Combining Remedio with Eyecatcher MD (AUC = 0.86) or iCare IOP (AUC = 0.83) also performed well. Portable devices were faster, had higher completion rates, and were strongly preferred by participants, particularly iCare and Eyecatcher.

**Conclusions:**

Portable devices showed good agreement with standard tools and high acceptability, supporting their potential role in glaucoma screening in resource-limited settings. Further community-based validation is recommended.

## Introduction

The global burden of glaucoma is rising, with prevalence projected to reach about 112 million people by 2040[1]. Disability-adjusted life years (DALYs) due to glaucoma have also increased significantly, with the burden being highest in low and middle sociodemographic index (SDI) regions[2]. In West Africa, countries such as Ghana, Guinea, Nigeria, Niger and Mali have some of the highest glaucoma prevalence rates. Mali recorded the highest rate at 351 per 100,000, while Nigeria followed with 309 per 100,000[3]. In a national survey conducted in Nigeria, glaucoma was identified as the second leading cause of blindness, responsible for 16.7% of all cases. Primary open-angle glaucoma (POAG) made up 86% of the diagnoses, with an overall prevalence of 5.02% among adults aged 40 years and older[4]. Additionally, one in five people with glaucoma was found to be blind, with visual acuity worse than 3/60 in the better eye[4]. The Tema Eye Survey (a population-based survey) in Ghana reported a 6.8% prevalence of POAG, with only about 3% of affected individuals being aware of their condition[5].

Population-based studies have reported varying prevalence rates across different regions and settings in India. The Chennai Glaucoma Study found a prevalence of 3.51% for POAG in an urban South Indian population aged 40 years and above[6]. A major challenge in addressing glaucoma in India is the high rate of undiagnosed cases which has been reported to be up to 90% in rural parts of India. Lack of adequate eye care facilities and trained ophthalmologists makes routine eye examinations and early detection challenging [6, 7].

Late diagnosis is a major contributor to glaucoma-related visual impairment and blindness in Africa and other low- and middle-income countries (LMICs)[4, 8–10]. Although the processes involved in glaucoma detection and management are complex, many high-income countries have accessible infrastructure in place to identify the disease at a pre-symptomatic stage, allowing timely intervention to prevent vision loss. In contrast, LMICs, particularly in sub-Saharan Africa, face a critical shortage of ophthalmologists and trained allied eye care personnel, with only 1.1 to 4.4 ophthalmologists per million people, compared to 76.2 per million in high income countries[2]. This gap is further exacerbated by limited access to modern standard diagnostic tools, hindering efforts to achieve early diagnosis and effective glaucoma management [4, 8, 11].

Portable devices have emerged as valuable tools and significantly advancing diagnosis and disease monitoring across various fields. The development of tools such as glucometers and blood pressure monitors has transformed health care delivery[12]. In ophthalmology, the introduction of portable devices capable of assessing visual function, measuring intraocular pressure (IOP), and capturing detailed images of the optic nerve and retina offers a promising complement to traditional diagnostic tools. Their potential for improving glaucoma diagnosis and monitoring is particularly relevant in low resource settings, where they may enhance access to essential eye care services. Devices such as the iCare tonometer are already in widespread use in research, screening, and clinical environments for IOP measurement. More recently, portable technologies are being explored for self-monitoring by glaucoma patients at home. These tools are generally user friendly, require minimal maintenance, and many are battery operated and compact enough to be stored in drawers or pouches[12–15].

Considering these challenges, there is a pressing need to explore alternative approaches to glaucoma diagnosis and monitoring. Portable devices, which are increasingly being adopted to assess key clinical parameters, offer a promising option where standard diagnostic equipment is inaccessible. However, their actual performance in real-world settings remains uncertain. This project therefore seeks to address basic but essential questions: how well their outputs agree with established reference measures, their diagnostic accuracy and how acceptable such devices are to patients.

Objectives of this study:

1. To compare selected portable assessment tools with standard reference tests in a hospital-based ophthalmology clinic.
2. To determine which individual test or combination of portable tests most closely matches a diagnosis of possible glaucoma based on standard diagnostic methods.

## Methods

This was a single site, cross sectional, observational, instrument method agreement study conducted at the Eye Clinic of University of Abuja Teaching Hospital (UATH), Abuja, Nigeria. Ethical approval for the study was obtained from the Health Research Ethics Committee of UATH (UATH/HREC/PR/149) and London School of Hygiene and Tropical Medicine (LSHTM Ethics Ref: 26813). We have used the sample size guide for binary logistic prediction models proposed by van Smeden et al [16], which suggests, given this number of predictors and prevalence of the outcome a sample size of 260 would be sufficient if the prediction error, measured using the root mean square percentage error, was 0.07. Therefore, to allow for some missing data we propose a sample size of 300 participants for the study.

## Study participants

### Inclusion criteria

Adult ( ≥ 18 years) patients attending the Eye Clinic of UATH were recruited for the study. This is a general outpatient clinic that sees approximately 300 patients each week with a wide range of eye conditions, including refractive errors, cataract, glaucoma, corneal, oculoplastic and retinal diseases. This allowed for a mix of patients with and without glaucoma, enabling an evaluation of how the portable devices compared with the existing standard reference methods for diagnosing glaucoma. All patients attending the clinic and accompanying persons who were willing to participate were included.

### Exclusion criteria

- Congenital or paediatric glaucoma.
- Visual acuity worse than LogMAR 1.00 (6/60) in the better-seeing eye from whatever cause: reduced vision would limit the ability to fixate and perform reliable tests, such as visual field assessments.
- Presence of corneal oedema: this prevents adequate visualisation of the posterior segment and would compromise the accuracy of optic disc imaging.
- Hospital inpatients.

### Study procedure

Patients and accompanying persons in the waiting area attending their clinic appointments were invited to participate in the study. Tests and examinations were organised in a sequential manner, with participants moving through a series of stations. Each station was managed by a research assistant, and completed tests were recorded or ticked as appropriate. All instructions were given in English. Where a participant could not understand English, instructions were provided in the preferred language whenever possible to support clear understanding of the procedures.

**Fig. 1:**
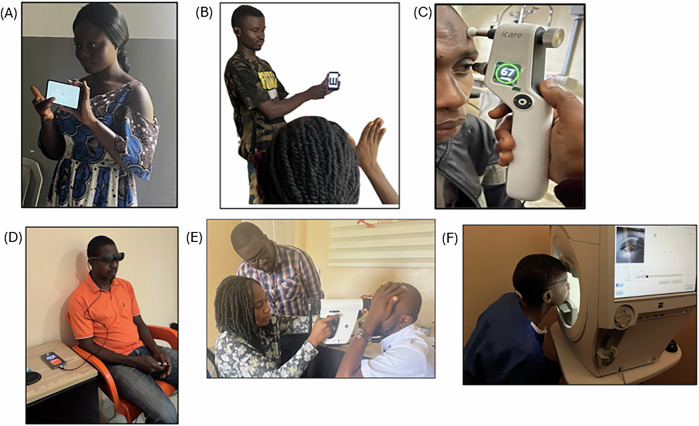
Pictures of portable devices in use. **A** PEEK VA, **B** PEEK CONTRAST, **C** iCare tonometer, **D** Eyecatcher visual Field analyser, **E** Remidio handheld fundus camera, **F** Humphrey Visual Field Analyser.

Portable devices and the corresponding standard diagnostics tests (Fig. 1) included;

- PEEK acuity app vs LogMAR chart.
- PEEK Contrast Sensitivity app vs Pelli-Robson test.
- Eyecatcher virtual reality (VR) headset-based perimeter and the Humphrey Field Analyzer (HFA).
- Remidio hand-held fundus camera vs Slit lamp fundoscopy using a 78D Volk Lens.
- iCare ic100 rebound tonometer vs Goldmann Applanation Tonometry (GAT).

#### Station 0—consent and registration

At the initial station, participants were welcomed and provided with information about the study. Written informed consent was obtained by trained research personnel, who explained the purpose, procedures, voluntary nature of participation, and the use of clinical images for research and publication purposes where applicable. Consent to publish was also obtained for any identifiable patient photographs. Once consent was obtained, participants were registered and issued a data collection form, which they carried with them throughout the subsequent testing stations.

#### Station 1—visual acuity

Visual acuity was assessed using two methods: a standard Tumbling E-LogMAR chart (Good-Lite Co, Elgin, Illinois, USA) and the PEEK Acuity smartphone application (Peek Vision Ltd, London, UK). The LogMAR chart was positioned at 4 m from the seating position. Participants reported the direction of each letter with their hands while covering one eye at a time. The PEEK Acuity test was conducted using a smartphone held at testing distance of 1 m. Similar tumbling E settings were selected for the PEEK Acuity app. Both tests were administered under standard room lighting conditions, and results were documented on the data collection form.

#### Station 2—contrast sensitivity

Monocular contrast sensitivity was assessed using the Pelli-Robson chart, and the PEEK Contrast Sensitivity app installed on a Sony Xperia Z3 smartphone. The Pelli-Robson test was conducted at a distance of one metre in a well-lit room, with participants reading the letters until they could no longer distinguish them. The PEEK Contrast Sensitivity test required participants to identify letters of decreasing contrast on the mobile screen.

#### Station 3—visual field assessment

Participants underwent visual field testing using both the Eyecatcher v3 smart glasses perimeter and the Humphrey Field Analyzer 3 (HFA). For devices testing was monocular, with the HFA testing each eye sequentially, while Eyecatcher interleaved the two eyes (i.e., with the target on each trial delivered to a single random eye). Both assessments took place in a dimly lit room, with participants instructed to fixate on a central target and press a button when they perceived a stimulus. Both devices produced single a Mean Deviation score for each eye, in dB. For Eyecatcher, the data were rescaled to be in the same dB units as the HFA, and used age-corrected normative data when computing MD[17, 18].

#### Station 4—optic disc photography/assessment

Optic disc images were captured using the compact Remidio non-mydriatic hand-held fundus camera (Remidio Fundus on phone, FOP NM 10). This smartphone-based fundus camera incorporates infrared imaging technology. It can be used as a handheld device, mounted on a slit lamp, or placed on its dedicated table stand for flexible operation. The system offers a 40° field of view and features integrated AI that can assist in glaucoma detection and provide recommendations for referral and further assessment. Participants were seated comfortably and asked to fixate on a target while the operator captured images of the optic nerve head. Images were taken for both eyes. The glaucoma AI and decision support system on the device was used to assess the vCDR of each eye which was stored on the phone for future retrieval[19]. Their reference for comparison was a slit lamp examination (see Station 6, below).

#### Station 5—tonometry

Intraocular pressure was measured using the iCare ic100 rebound tonometer. Each participant’s eye was assessed without the use of anaesthetic drops. The device automatically captured six readings per eye and calculated an average value, which was recorded in a separate book. There reference for comparison was GAT (see Station 6, below).

#### Station 6—slit lamp examination

A consultant ophthalmologist (Principal Investigator), masked to the results of all prior tests, conducted a comprehensive slit lamp examination. This included;

- Assessment of anterior and posterior segments of the eyes
- Gonioscopy using a four mirror gonioscopy lens to assess the anterior chamber angle and graded with Shaffer’s grading system.
- Goldmann applanation tonometry was performed, with one to three readings taken during the same sitting. While adjusting the tonometer, the principal investigator also read the measurements. The average of these readings was recorded.
- Fundoscopy was performed using a 78D Volk lens at the slit lamp to examine the optic nerve head. The vCDR was estimated by comparing the vertical cup diameter to the vertical disc diameter.

After completing all the stations, participants were asked to comment on their experience and satisfaction with the portable devices and how they felt about having these tests done again as part of their routine clinic follow up.

### Study outcome measures

The principal outcome measures were:

- Correlations and levels of agreement between portable diagnostic tests and their respective standard reference methods.
- Identification of the individual portable test or combination of tests demonstrating the highest diagnostic accuracy, measured by sensitivity and specificity for detecting glaucoma.

Secondary outcome measures included:

- Time taken to conduct each test
- Completion rate of test (Example Humphrey & Eyecatcher). This refers to the proportion of tests that ran from start to finish and produced a device specific reliability criteria and analysable results.
- Patient’s preference for tests conducted using portable devices vs standard reference tests.

### Statistical analysis

Pearson’s coefficients were calculated to compare correlation between portable and standard equipment. Bland–Altman plots were used to measure agreement. ROC curves were plotted to display sensitivity and specificity of devices in the diagnosis of glaucoma and cut off points. STATA 18.1(Texas, USA) was used to conduct the statistical analyses. *P-*values of less than 0.05 were considered statistically significant.

Predefined levels of agreement guided interpretation of the correlation and Bland–Altman analyses. The correlation coefficient (r), which measures how closely two variables move together, was classed as strong when 0.70 or higher, moderate when between 0.40 and 0.69, and weak when below 0.40. Bland–Altman agreement was very good when the mean difference was close to zero with over 95 percent of values within narrow limits. Good agreement referred to slightly wider but clinically acceptable limits, while moderate agreement described broader limits suitable for screening or triage. Poor agreement indicated limits wide enough to affect clinical interpretation[20, 21].

## Results

Table 1 presents the demographic characteristics of the study participants. A total of 312 individuals were recruited, contributing 524 eyes for analysis. The mean age was 44 years (SD ± 14.44), with a range of 18 to 95 years and a median age of 42 years. The largest age group was 40–49 years (28.1%). Female participants accounted for 57.4% of the sample. Most had tertiary education (69.6%), while only 2.2% had no formal education.

**Table 1:** Demographic Characteristics of Study Participants (*N* = 312).

Characteristic	Category	Frequency (*n*)	Percent (%)
Age Group (years)	18–29	53	17.0
	30–39	63	20.2
	40–49	89	28.5
	50–59	65	20.8
	60–69	27	8.7
	≥70	15	4.8
Sex	Female	179	57.4
	Male	133	42.6
Education Level	None	7	2.2
	Primary	14	4.5
	Quranic	4	1.3
	Secondary	70	22.4
	Tertiary	217	69.6

**Fig. 2:**
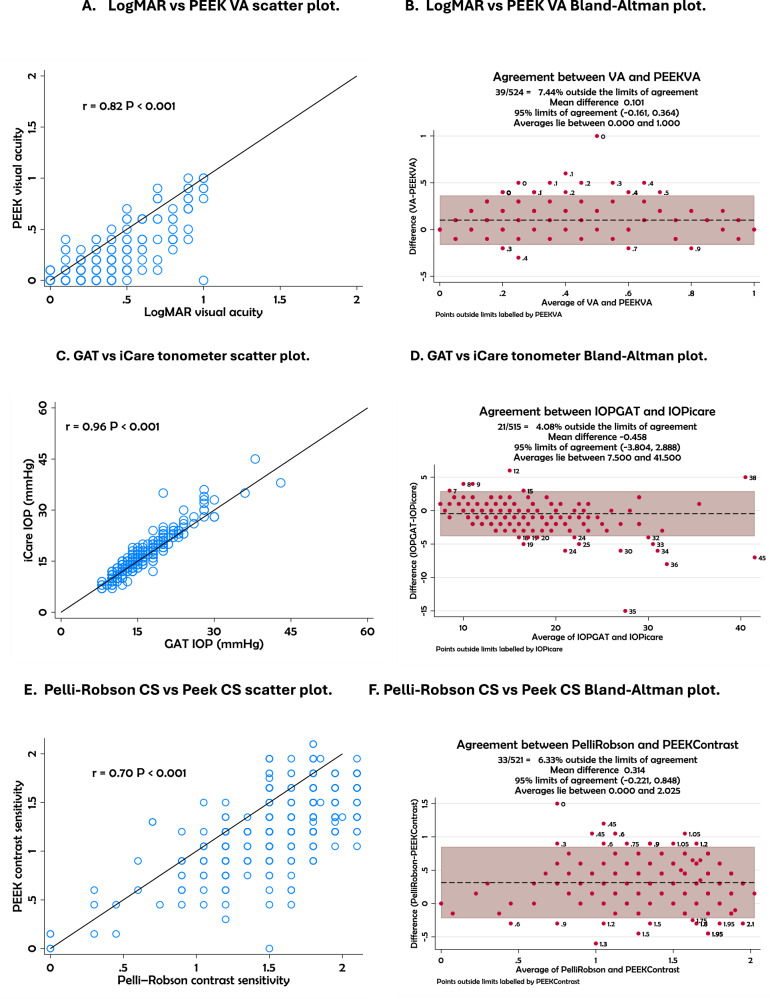

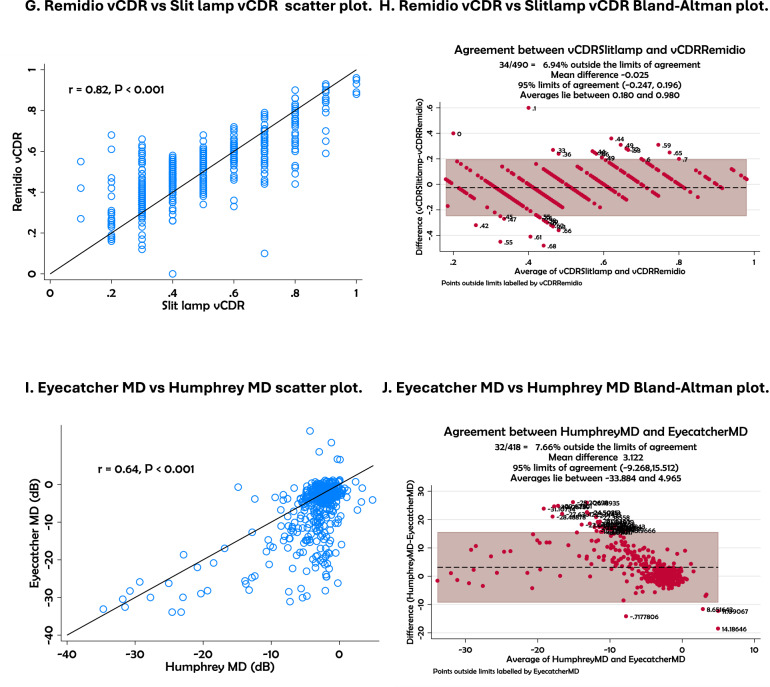
Scatter plots and Bland–Altman plots for tests. **A**, **B** LogMAR vs PEEK VA; **C**, **D** GAT IOP vs iCare IOP; **E**, **F** Pelli-Robson CS vs Peek Contrast Sensitivity; **G**, **H**, Remidio CDR vs Slit lamp CDR; **I**, **J** Eyecatcher MD vs HFA MD.

The scatter plots and Bland–Altman (Fig. 2) analyses demonstrate varying levels of agreement between portable and standard diagnostic tools for glaucoma assessment. Strong positive correlations were observed for LogMAR versus Peek VA (*r* = 0.82), GAT versus iCare tonometer (*r* = 0.96), and Remidio versus slit lamp CDR assessments (*r* = 0.82), all with *p* < 0.001. Moderate correlations were found for Pelli Robson CS versus Peek CS (*r* = 0.70) and Eyecatcher versus HFA mean deviation (MD) (*r* = 0.64), also with *p* < 0.001.

The Bland–Altman plots showed acceptable agreement across these comparisons. For visual acuity, 7.47% of data points were outside the limits of agreement, with values distributed between 0.0 and 1.0. IOP measurements using GAT and iCare showed a mean difference of −0.488 mmHg, with only 4.31% of points outside the 95% limits (−3.896 to 2.920). In contrast sensitivity, Pelli Robson and Peek CS differed by a mean of 0.313, with limits ranging from -0.222 to 0.848 and 6.48% of values outside the range. For optic disc imaging, Remidio and slit lamp CDR values showed a mean difference of −0.028, with 7.06% outside the 95% agreement range (−0.264 to 0.189). Eyecatcher versus HFA MD comparison showed a broader range of agreement (−9.148 to 5.236) with 7.39% of points outside the limits and a mean difference of 3.044. Agreement between vCDR measurements from the slit lamp and the Remidio fundus camera was substantial with quadratic-weighted Cohen’s kappa (κ = 0.79, 95% CI 0.74–0.84) and almost perfect with Gwet’s AC (AC = 0.92, 95% CI 0.89–0.94). These statistics were used to assess how closely the two methods classified optic disc measurements. Cohen’s kappa adjusts for the amount of agreement expected by chance, but it can be affected by uneven category distribution, which may lower the value even when overall agreement is high.

Glaucoma diagnosis was made using the criteria listed below, and both (i and ii) had to be met.

i. Slit lamp vCDR ≥ 0.8. This was derived from the Nigeria normative data for defining glaucoma in prevalence surveys[22].
ii. HFA Mean Deviation (MD) ≥−6.00 dB. This was also derived from categorising the stage of glaucoma from pre-diagnosis to end-stage disease[23], which classified Early Glaucoma MD as ≥−6.00 dB and at least one of the following;

- On pattern deviation plot, there exists cluster of 3 or more points in an expected location of the visual field depressed below the 5% level, at least 1 of which is depressed below the 1% level
- Corrected pattern standard deviation/pattern standard deviation significant at *P* < 0.05
- Glaucoma hemifield test “Outside Normal limits”

Intraocular pressure (IOP) was not used as a diagnostic criterion because participants already diagnosed with glaucoma were on IOP-lowering treatment, which would have affected measured values. Using these criteria, glaucoma prevalence in the sample was 5.92%.

**Fig. 3:**
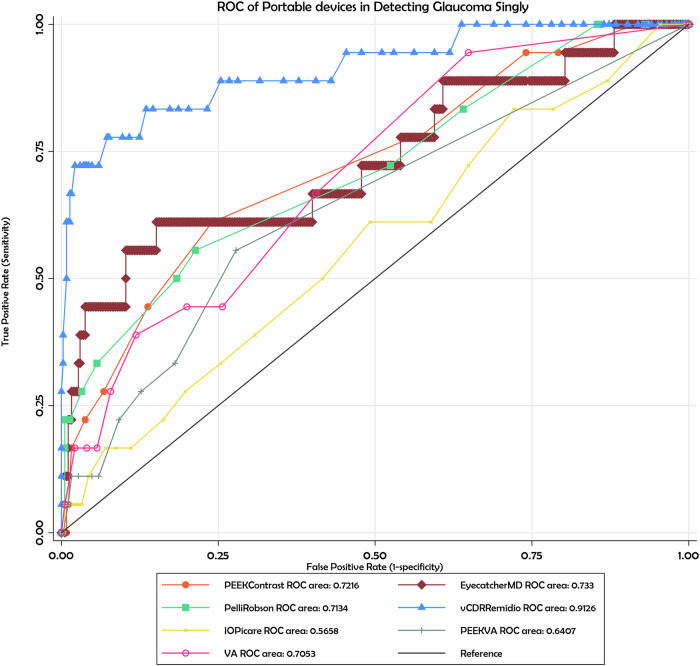
ROC Analysis of Individual Portable device in Glaucoma Detection.

Receiver Operating Characteristic (ROC) analyses (Fig. 3, Table 2) were performed to assess the diagnostic performance of individual portable devices for glaucoma detection when compared with standard tests and slit-lamp vCDR. Among the tests, the Remidio fundus camera measuring vertical cup-to-disc ratio (vCDR) demonstrated the highest accuracy with an AUC of 0.9126. At a cut-off value of vCDR ≥ 0.79, it achieved a sensitivity of 82.35% and specificity of 99.77%, correctly classifying 99.12% of eyes. Eyecatcher (Mean Deviation) showed moderate accuracy with an AUC of 0.7330, sensitivity of 64.71% and specificity of 92.02%. The iCare tonometer had an AUC of 0.5658, sensitivity of 85.71% and specificity of 95.83% at ≥25 mmHg. PEEK Visual Acuity (VA) and Contrast Sensitivity (CS) showed lower AUC values of 0.6407 and 0.7216 respectively, with PEEK CS demonstrating no sensitivity at the selected cut-off ( ≥ 2.1) despite high specificity (94.97%). The optimal cut-off for PEEK CS of ≥2.1 is a higher threshold than expected from Pelli–Robson values. This reflected a ceiling effect in the distribution of PEEK CS scores, which shifted the optimal point on the ROC curve towards the upper end of the scale. At this threshold, sensitivity was zero, indicating that contrast sensitivity did not distinguish glaucomatous from non-glaucomatous eyes in this sample.

**Table 2:** Area Under Curve (AUC), Sensitivity and Specificity of portable devices to detect glaucoma individually or in combination.

Portable Device (Singly or in combination)	AUC	Cut off (>=)	Correctly classified %	Sensitivity	Specificity
Remidio fundus camera vCDR	0.9126	0.79	99.12%	82.35%	99.77%
Eyecatcher MD	0.7330	−20.09 dB	90.91%	64.71%	92.02%
Remidio vCDR + ICare IOP	0.8299	Yes glaucoma	98.64%	66.67%	99.31%
Remido vCDR + Eyecatcher MD	0.8554	Yes Glaucoma	93.86%	76.47%	94.62%
Eyecatcher MD + ICare IOP	0.7944	Yes Glaucoma	98.35%	60.00%	98.88%

Combinations of portable tests were also evaluated. The pairing of Remidio vCDR and Eyecatcher MD produced an AUC of 0.8554, with sensitivity of 76.47% and specificity of 94.62%. Combining Remidio vCDR with iCare IOP yielded an AUC of 0.8299, with sensitivity of 66.67% and specificity of 99.31%. The combination of Eyecatcher MD and iCare IOP achieved an AUC of 0.7944, with sensitivity of 60.00% and specificity of 98.88%. These findings suggest that combining structural (optic disc) and functional (visual field) measures enhances the diagnostic utility of portable devices, particularly in settings where standard equipment is inaccessible.

## Discussion

The Remidio handheld fundus camera is a portable option of assessing the optic disc. It integrates Medios Glaucoma AI, which operates entirely offline to analyse the optic disc characteristics and retinal nerve fibre layer (RNFL) thinning. Based on those features (vCDR and RNFL defects), the AI generates a referral recommendation identifying individuals with signs suggestive of glaucoma who require further assessment. In clinical validation studies, this system demonstrated strong diagnostic performance, achieving sensitivity of 91–94% and specificity of 85–94% in detecting referral-warranted glaucoma compared to specialist evaluation[19]. Assessment of vCDR with Remidio handheld fundus camera when compared with conventional slit lamp with a 78D lens showed a strong correlation in this study (*r* = 0.80) and a close 95% limits agreement of between 0.264 and 0.189. Remidio handheld fundus camera has been compared with other devices like Pictor and they achieved comparable results to the Zeiss table-top camera[19].

The Remidio handheld fundus camera vCDR demonstrated excellent overall diagnostic performance. An AUC of 0.9126 indicates strong ability to distinguish between glaucomatous and non-glaucomatous eyes, and the optimal cut-off of ≥ 0.79 produced very high accuracy (99.12% correctly classified). Specificity was particularly high (99.77%), indicating the device is highly reliable in identifying healthy eyes and reducing false positives, which is advantageous for community screening and avoiding unnecessary referrals. Sensitivity (82.35%), while good, was lower than specificity, indicating that a small proportion of true glaucoma cases could be missed. This trade-off is typical in devices prioritising specificity and highlights the importance of follow-up for borderline or suspicious findings. When combined with other portable tools, performance remained strong. The combination of Remidio vCDR with iCare IOP achieved an AUC of 0.8299, with sensitivity of 66.67% and specificity of 99.31%. Similarly, pairing Remidio vCDR with Eyecatcher MD yielded an AUC of 0.8554, with sensitivity of 76.47% and specificity of 94.62%. The Eyecatcher MD and iCare IOP combination recorded a slightly lower AUC of 0.7944, but maintained excellent specificity (98.88%) and acceptable sensitivity (60.00%). These findings imply that while combined portable parameters maintain high specificity and offer good overall diagnostic performance, their discriminative ability is slightly lower than the Remidio vCDR alone. Majority of the participants (97%) in this study showed preference to Remidio handheld fundus camera as a tool for assessing the optic disc, compared to only 1% who preferred the standard slit lamp examination. Consequently, Remidio handheld fundus camera could be a valuable tool for the screening of glaucoma in low resource communities where acquiring and managing multiple tools could be a challenge as other non-mydriatic portable devices have been used with promising results[24].

The HFA is a large, technically complex device that requires careful handling and the involvement of trained personnel to ensure accurate testing and maintenance. Test takers must sit in a certain position, fixing their eyes on a central target while concentrating on seeing flashing lights of different intensities in their field of view and responding appropriately (Fig. 1F). This makes it more challenging especially for individuals with significant visual impairment or problems with posture. The Eyecatcher is a portable VF assessing device comprising of an android phone, a virtual reality goggles and a clicker (Fig. 1D). Test takers sit comfortably on a chair or sofa wearing the goggles connected to the phone and holding the clicker. Earlier versions, such as Eyecatcher 2.0, were designed to run on a tablet or laptop and used an external eye patch to test each eye separately. These earlier versions offered a larger screen and were validated for home monitoring of glaucoma, but patients reported practical drawbacks such as bulkiness, the need to control viewing distance and lighting, and difficulties with patching and data transfer, which led to the development of the lighter and more compact smart glasses system[17]. Other principles of the Eyecatcher are similar to HFA. All participants in this study expressed a preference for Eyecatcher regarding ease of use and comfort and a similar observation was made by Nida et al.[25], and Phu J et al.[26] with a similar device. Analysis of results from both devices however shows moderate correlation (*r* = 0.66) which is statistically significant (*p* < 0.001). The high proportion of participants with university level education (67%) in this study may limit how well the findings apply to the wider population. People with higher education may find visual field testing easier to understand and complete, which can improve test reliability. This demographic profile should be considered when interpreting the results.

The portable iCare tonometer has improved the measurement of intraocular pressure as it does not require anaesthetic drops or fluorescein dye, and is quicker and more comfortable than GAT. In this study, IOP measurements from iCare and GAT showed very strong correlation (*r* = 0.96), which is consistent with several published comparisons reporting similar agreement between the two methods[27–31]. However, variations between the two measurements were noticed more at extreme readings which was also reported by Gao et al. [27] where they recorded lower IOP values with iCare when GAT ≥ 23 mmHg.

In this study, patients with pre-existing glaucoma diagnosis were on treatment which influenced the IOP readings. Hence, making the role of iCare in diagnosing glaucoma difficult to determine. This affected the ROC analysis, producing a low AUC even though sensitivity and specificity were high at the chosen cut-off. The threshold of 25 mmHg still performed well because very few non-glaucomatous eyes reached this level, while some untreated or poorly controlled glaucoma cases remained above it. This reflects treatment effects rather than true diagnostic ability and shows that IOP was not suitable as a standalone screening test in this sample. iCare IOP was included in the ROC analysis to demonstrate its agreement with GAT in a clinic setting, and the findings support its use as a reliable method for measuring IOP rather than diagnosing glaucoma. Its value in community care remains important, as it can detect raised untreated IOP. The strong agreement with GAT supports its use in primary or outreach settings alongside other assessments to guide referral. The portability of iCare also makes it easier to use in elderly or disabled patients, and it can be better tolerated by individuals who struggle with GAT, sometimes due to blepharospasm during applanation[27, 32].

The findings for visual acuity and contrast sensitivity were also notable. The strong correlation between LogMAR and the PEEK visual acuity app indicates that portable acuity testing can provide results that closely match standard clinic-based assessment. In contrast, the performance of the PEEK contrast sensitivity test was much weaker. The compressed distribution of scores and the ceiling effect in this sample limited its ability to distinguish between glaucomatous and non-glaucomatous eyes, reflected in the very low sensitivity observed in the ROC analysis. These results suggest that while portable visual acuity assessment has potential clinical value, portable contrast sensitivity testing using PEEK contrast sensitivity in its current form is unlikely to contribute meaningfully to glaucoma case finding.

The Completion rates were high across most tests. The iCare tonometer achieved a 99% completion rate, while the slit lamp (78D) and Remidio handheld fundus camera were completed by 98% and 97% of participants, respectively. Slightly lower rates were recorded for the Eyecatcher visual field test (93%), the Humphrey visual field analyser (89%), and the GAT (86%), which may reflect the greater complexity or discomfort associated with these procedures.

The level of agreement across the portable tests relates directly to their clinical usefulness. Strong agreement from the Remidio camera supports its role in clinical decision making, while iCare provides reliable IOP measurement but cannot guide decisions on its own. Moderate agreement, as seen with Eyecatcher, suggests a role in screening or triage rather than diagnosis. Tests with weaker agreement offer limited diagnostic value but may still support broader assessment. The portable devices also offer practical advantages with meaningful public health implications. These devices are compact and robust and operate without mains power, which allows their use in community settings and reduces the need for patients to travel to urban hospitals. This can lessen indirect costs of care, including loss of income and transport expenses, which often act as barriers to access. Their relatively low cost and the feasibility of use by trained non ophthalmologists increase their suitability for screening programmes in rural or underserved areas. Several of the tools assessed in this study can also detect other ocular diseases, which broadens their value in routine eye care. For example, the Remidio handheld fundus camera includes an AI function that can identify diabetic retinopathy, and visual field testing can reveal neurological field loss. These strengths position portable devices as practical options for improving early glaucoma detection and support future development of scalable comprehensive community based eye care screening models.

## Conclusions

This study demonstrates that portable diagnostic devices, including the Remidio handheld fundus camera, Eyecatcher visual field system, and iCare tonometer, can achieve good to excellent agreement with gold-standard hospital-based equipment for the assessment of structural and functional parameters in glaucoma. While sensitivity varied between devices and was generally lower than specificity, their performance was sufficient to identify a substantial proportion of individuals warranting further evaluation. The Remidio handheld fundus camera, in particular, showed excellent diagnostic accuracy and strong participant preference, while the iCare tonometer demonstrated high completion rates and good agreement with Goldmann applanation tonometry.

Beyond diagnostic metrics, these devices offer practical advantages that are highly relevant to glaucoma case-finding in resource-limited settings. Their portability, robustness, and independence from mains electricity make them well suited for deployment in community-based screening, thereby reducing the need for patients to travel to urban centres, take time off work, and incur associated costs. Their relatively low cost and suitability for use by trained non-ophthalmologists further enhance their scalability. Taken together, these findings support the integration of such portable technologies into community eye health strategies, potentially enabling earlier detection and intervention for glaucoma in underserved populations.

### Study limitations

- The study was conducted in a controlled or semi-controlled environment; performance, completion rates, and usability may differ under true field conditions in rural or resource-limited settings.
- Diagnosed glaucoma participants receiving treatment, particularly IOP-lowering therapy, may have limited the utility of IOP as a determinant for glaucoma diagnosis in this study and influenced the observed agreement between devices.
- Some participants have been previously exposed to the standard diagnostic tests, which could have influenced their performance, completion rates, or reported preferences for certain devices.

## Summary

### What was known before?

- Glaucoma causes substantial avoidable blindness in low and middle income countries, largely due to late diagnosis.
- Standard glaucoma diagnostics require specialist equipment, trained staff, and stable power supply.
- Portable eye tests exist, but evidence on their diagnostic accuracy and agreement with gold standard tests in real clinical settings is limited.

### What this study adds?

- The Remidio handheld fundus camera demonstrated very high diagnostic accuracy for detecting referable glaucoma.
- Combining portable structural and functional tests improved diagnostic performance.
- Portable tests were quicker to perform, had high completion rates, and were strongly preferred by patients.
- These findings support the use of portable devices for glaucoma case finding in resource limited settings.

## Supplementary information

eye-reporting-checklist

## Data Availability

The datasets generated and analysed during this study are available from the corresponding author on reasonable request. All relevant raw data will be made available for non commercial research use in accordance with Springer Nature Data Policy Type 3, subject to approval by the relevant ethics committees and in a manner that does not compromise participant confidentiality.
